# Analysis of endometrial liquid‑based cytology samples to detect somatic mutations and classify ovarian cancer

**DOI:** 10.3892/ol.2025.14866

**Published:** 2025-01-07

**Authors:** Michiko Kubo-Kaneda, Eiji Kondo, Ryo Nimura, Kota Okamoto, Tsuyoshi Matsumoto, Kenta Yoshida, Makoto Ikejiri, Maki Nakamura, Hiroshi Imai, Yoshinaga Okugawa, Kaname Nakatani, Tomoaki Ikeda

**Affiliations:** 1 Department of Obstetrics and Gynecology, Mie University School of Medicine, Tsu, Mie 514-8507, Japan; 2 Department of Gynecologic Oncology, Cancer Institute Hospital, Tokyo 135-8550, Japan; 3 Department of Genomic Medicine, Mie University School of Medicine, Tsu, Mie 514-8507, Japan; 4 Department of Oncologic Pathology, Mie University School of Medicine, Tsu, Mie 514-8507, Japan

**Keywords:** cytology, ovarian cancer, next-generation sequencing, mutation

## Abstract

Ovarian cancer has a poor prognosis, and screening methods have not been established. Biomarkers based on molecular genetic characteristics must be identified to develop diagnostic and therapeutic strategies for all cancer types, particularly ovarian cancer. The present study aimed to evaluate the usefulness of genetic analysis of cervical and endometrial liquid-based cytology (LBC) specimens for detecting somatic mutations in patients with ovarian cancer. The data of 19 patients with ovarian cancer treated between August 2019 and July 2022 were analyzed. LBC specimens from the cervix and endometrium of patients with preoperatively suspected ovarian cancer were collected, and genomic DNA was extracted from these LBC specimens and surgically removed cancer tissue sections for genetic analysis. Next-generation sequencing (NGS) analysis of cervical and endometrial LBC revealed genetic mutations similar to those in formalin-fixed, paraffin-embedded (FFPE) tissues in 42% of ovarian cancer cases, including negative cervical and endometrial cytology cases and early-stage cases. The pathogenic variants detected were *PIK3CA* (n=1), *RB1* (n=1) and *TP53* (n=6). In high-grade serous carcinoma (HGSC) cases, the diagnosis rate was 54.5%, which was higher than that of other histological types. In univariate analysis of patients with HGSC, the presence of serous tubal intraepithelial carcinoma tended to be associated with the detection of somatic mutations in LBC samples. NGS analysis of cervical and endometrial LBC samples revealed genetic variants similar to those in FFPE tissues from ovarian cancer cases and may be useful as a noninvasive screening method for detecting somatic mutations and classifying ovarian cancer.

## Introduction

The proportion of patients diagnosed with ovarian cancer is increasing worldwide. In 2021, approximately 19,710 women were diagnosed with ovarian cancer, among whom 13,270 died due to the disease (1). Approximately 57% of patients with ovarian cancer are diagnosed with metastasis, and the 5-year survival rate is 50.8%, which is an extremely poor prognosis (1). Large cohort studies using cancer antigen 125 (CA125) testing and transvaginal ultrasonography as early screening methods have been conducted in the UK (2,3); however, none could prove their efficacy. Similar studies have been conducted on patients at a high risk of ovarian cancer; however, while serum CA125 testing and transvaginal ultrasonography are options, whether they improve survival in screened high-risk women remains unclear (4).

Magnetic resonance imaging (MRI) is a useful tool for identifying ovarian tumors (5). For ovarian mass assessment, the Ovarian-Adnexal Reporting Data System MRI (O-RADS MRI) score, which assesses the perfusion of solid tissue using a time-intensity curve with the myometrium used as the internal reference, is used (6). Validation of the O-RADS MRI score showed a sensitivity and specificity of 93 and 91%, respectively, for the detection of malignant lesions in masses that were undetectable on ultrasound, regardless of the level of radiology expertise. However, several cases of early-stage ovarian cancer are asymptomatic, and the O-RADS MRI score is utilized for the follow-up of ovarian masses.

In addition, 18-Fluoro-deoxyglucose (FDG) positron emission tomography imaging is unsuitable for the primary detection of ovarian cancer because of FDG uptake in the late follicular to early luteal cysts in premenopausal females and low FDG uptake in clear cell and mucinous invasive subtypes (7).

Therefore, surveillance methods for the early detection of ovarian cancer have not been established.

The main types of ovarian cancer are high-grade serous carcinoma (HGSC), endometrioid carcinoma, clear-cell carcinoma (CCC), low-grade serous carcinoma (LGSC), and mucinous carcinoma (MC), all with differing molecular mechanisms and carcinogenesis processes. Recently, genomic analysis of cancer samples from The Cancer Genome Atlas has revealed genes in all cancer types, leading to a decision to target genetic mutations (8).

Regarding ovarian cancer, CCC has few tumor protein 53 (*TP53*) mutations but frequent AT-rich interactive domain-containing protein 1A (*ARID1A*) and phosphatidylinositol-4,5-bisphosphate 3-kinase catalytic subunit alpha (*PIK3CA*) mutations, whereas HGSC has mostly *TP53* mutations (9,10). For HGSC, the presence of four transcriptional subtypes (immunoreactive, differentiated, proliferative, and mesenchymal) was confirmed using de novo classification (11). The efficacy of combination therapy with poly (ADP-ribose) polymerase inhibitors and bevacizumab in treating pathological breast cancer susceptibility gene (*BRCA*)-mutated and homologous recombination deficiency-positive ovarian tumors has been reported, with survival benefit observed in some cases (12,13). However, ovarian cancer has fewer indications for molecular-targeted agents than other carcinomas.

The European Society for Medical Oncology (ESMO) recommends the routine use of next-generation sequencing (NGS) in cases of advanced non-squamous non-small-cell lung cancer, prostate cancer, ovarian cancer, and cholangiocarcinoma based on current evidence. Because of the differences in their carcinogenesis processes, the histological types of ovarian cancer have different responses to chemotherapy (14). The ESMO-European Society of Gynaecological Oncology consensus conference recommends primary debulking surgery with no macroscopic residual disease for cases of LGSC, MC, and CCC due to the low chemosensitivity (15). To determine the optimal treatment strategy, identifying the histological type of ovarian cancer preoperatively is crucial.

Endometrial cytology has been reported as a diagnostic method for ovarian cancer (16). The appearance of tumor cells in the cytology specimens of the endometrium and cervix through the fallopian tubes results in positive endometrial cytology. Hirasawa *et al* (16) reported that 23.0% of patients with ovarian cancer had positive endometrial cytology specimens. Another study showed a detection rate of 45% for early-stage ovarian cancer based on genetic analyses of the DNA in 18 cancer-related genes recovered from the liquid biopsies obtained during a routine Papanicolaou test of the cervix and endometrium (17). Recently, the use of liquid-based cytology (LBC) specimens for NGS analysis has been reported (18).

Therefore, in the present study, we focused on cervical and endometrial LBC samples. Identifying biomarkers based on molecular genetic characteristics may contribute to developing diagnostic and therapeutic strategies for ovarian cancer. In this study, we aimed to examine the usefulness of a noninvasive screening method for ovarian cancer, including histological identification, by combining cervical and endometrial cytology and NGS analysis of LBC samples.

## Materials and methods

### Patients and clinical information

This study included 19 patients with ovarian cancer treated between August 2019 and July 2022 at the Department of Obstetrics and Gynecology, Mie University Hospital. First, we collected cervical and endometrial LBC specimens from patients with suspected ovarian cancer before surgery. For LBC processing, CelVerse TM (Sysmex Corporation), with an alcohol content of 40.5%, was used as the cell preservation solution according to the manufacturer's protocol, and cytologically diagnosed using light microscopy after Papanicolaou staining. Surgically resected organs were fixed in 10% buffered formalin at room temperature for 24 to 48 h, and the tissue specimens were formalin-fixed and paraffin-embedded using an automated tissue processor (Tissue-Tek^®^ VIP6; Sakura Finetek Japan Co., Ltd.). FFPE specimens were sectioned at 4 µm and stained with hematoxylin and eosin using an autostainer (Leica Autostainer XL).

Genomic DNA was extracted from the LBC specimens and cancer tissue sections that were surgically removed for genetic analysis. We evaluated the usefulness of the genetic analysis of LBC specimens for ovarian cancer by examining whether pathological genetic mutations detected in cancer tissue sections could also be detected in cervical and endometrial LBC specimens.

This study was approved by the Ethics Committee of Mie University Hospital (approval no. H2020-075) and was conducted according to the standards of the Declaration of Helsinki, revised in 2001.

### DNA extraction from LBC specimens and formalin-fixed, paraffin-embedded tissue

After reviewing the histopathological findings, formalin-fixed, paraffin-embedded (FFPE) tissue from the cancer site was serially sectioned at a thickness of 10 µm, mucosal tissues from each cancer region were microdissected, and genomic DNAs were extracted using the QIAmp DNA FFPE tissue kit (Qiagen, Valencia, CA, USA) according to the manufacturer's instructions. LBC specimens were refrigerated at 4°C, and genomic DNA was extracted from LBC specimens using QIAmp DNA Mini kits (QIAGEN) according to the manufacturer's instructions. The quality and quantity of each DNA sample were evaluated using the Qubit^®^ dsDNA or RNA HS Assay Kit (ThermoFisher Scientific, Waltham, MA, USA) according to the manufacturer's instructions.

### Next-generation sequencing analysis of 50 cancer-related genes

NGS analysis of genomic DNAs from each sample was performed using the Ion AmpliSeq Cancer Hotspot Panel v2, which covers approximately 2,800 mutational hotspot regions from 50 cancer-related genes, as previously described (19–21). Ovarian cancer has been reported to involve somatic mutations in common cancers from the Catalogue of Somatic Mutations in Cancer, including *TP53, PIK3CA*, Kirsten rat sarcoma viral oncogene homolog (*KRAS*), Catenin beta-1 (*CTNNB1*), and SWI/SNF related, matrix associated, actin dependent regulator of chromatin, subfamily a, member 4 (*SMARCA4*), which were partially covered in the present study (22). Genomic DNAs (10 ng) were extracted from FFPE or LBC specimens and used to construct barcoded DNA libraries using an Ion AmpliSeq Library Kit Plus (ThermoFisher Scientific). The libraries obtained were purified using the Agencourt AMPure XP Reagent (Beckman Coulter, Brea, CA, USA) and then sequenced using an Ion Personal Genome Machine or Ion S5 platform (ThermoFisher Scientific). The sequencing reads were aligned to the reference genome builds hg19 and GRCh37 and converted into binary alignment map files using Ion Torrent Suite software (ThermoFisher Scientific). Sequence variant calling was performed using Ion Reporter 5.12 (ThermoFisher Scientific) according to the manufacturer's instructions. The mean read depth of coverage in the DNA sequencing was >1,500-fold. Somatic mutations were classified as ovarian cancer mutations based on the following criteria: i) variant allele frequency of somatic mutations in tumor tissues >5%, ii) variant allele frequency of somatic mutations in LBC >0.1%, and iii) registration of mutations as ‘pathogenic/likely pathogenic variants’ according to the ClinVar database.

### Statistical analysis

All statistical analyses were performed using SPSS (version 28.0; IBM Corp., Armonk, NY, USA), and statistical significance was set at P<0.05. The hazard ratios and associated 95% confidence intervals were calculated using a stratified Cox proportional hazards model to identify the independent predictors of genetic mutation concordance between surgically removed cancer tissue sections and cervical and endometrial LBC specimens. Factors included in the univariate analyses included positive endometrial cytology, positive ascites cytology, positive lymph vascular invasion, presence or absence of serous tubal intraepithelial carcinoma (STIC), and presence or absence of peritoneal dissemination.

## Results

Clinical data are presented in Table I. The median age of the patients was 60 years (range: 34–80, and the mean body mass index was 21.3 kg/m^2^ (range: 17.4–30.4). The major complaints were abdominal distention (n=8), abdominal pain (n=4), constipation (n=1), ovarian tumor during follow-up (n=2), and anorexia (n=1). Three patients were asymptomatic and were diagnosed with advanced-stage ovarian cancer. The pathological data of the patients are presented in Table II. Adenocarcinoma was identified in one case of cervical cytology and three cases of uterine cytology.

**Figure 1.**
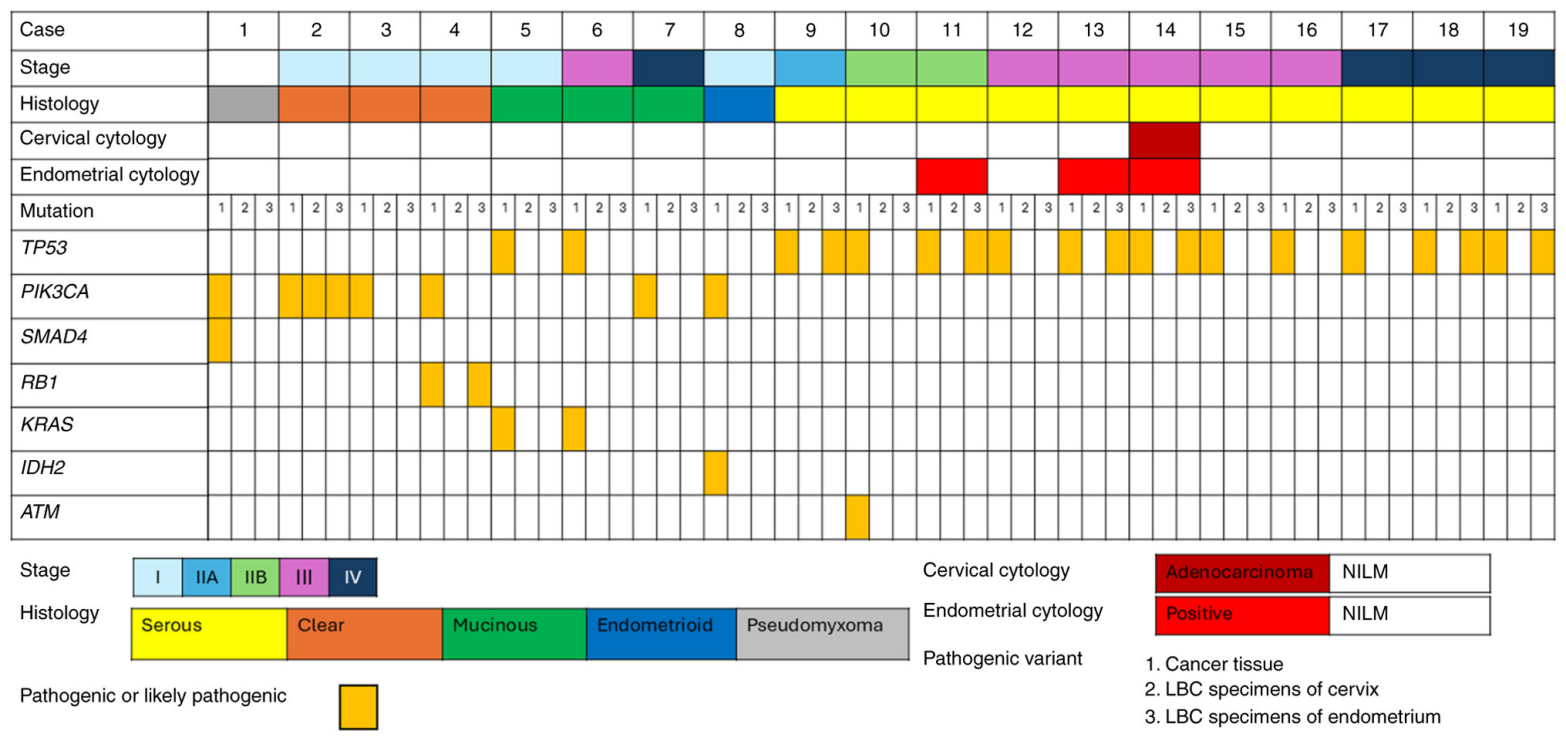
Pathogenic variants detected in cancer tissues and cervical and endometrial LBC specimens based on the World Health Organization stage classification, histology, cervical cytology and endometrial cytology. LBC, liquid-based cytology; NILM, negative for intraepithelial lesion or malignancy.

Pathogenic variants detected in cancer tissue sections and cervical and endometrial LBC specimens are shown in Fig. 1. Pathogenic variants detected in cancer tissue sections were found in endometrial LBC specimens from eight patients and a cervical LBC specimen from one patient. Variants of matching genes are listed in Table III. The following pathogenic variants were detected: *PIK3CA* (n=1), *RB1* (n=1), and *TP53* (n=6).

Cervical and endometrial LBC specimens were collected preoperatively from patients with a preoperative diagnosis of ovarian cancer; however, the specimen was collected from one patient after preoperative chemotherapy (Case 14), and cervical and endometrial cytology revealed the presence of adenocarcinoma. Notably, no pathogenic variants were identified in the cervical LBC specimens. Five of the eight patients with matching pathogenic variants were negative for malignancy on cervical and endometrial cytology. Three of the eight patients with matching pathogenic variants in the LBC specimens were at an early stage (stage 1C1, 1C2, or 2A).

The rate of genetic analysis concordance between endometrial LBC specimens and cancer tissue sections was 42%. In HGSC cases, the concordance rate was 54.5%. In the univariate analysis, no factor was associated with the concordance between cancer tissue sections and cervical and endometrial LBC specimens (Table IV). In the univariate analysis of patients with HGSC, the presence of STIC was associated with concordance between cancer tissue sections and LBC specimens (Table V).

## Discussion

The present study revealed that NGS analysis of cervical and endometrial LBC samples revealed genetic mutations similar to those in FFPE tissue in 42% of ovarian cancer cases, including negative cervical and endometrial cytology cases and early-stage cases. Therefore, NGS analysis of endometrial LBC samples may be a useful screening method for ovarian cancer.

Notably, several studies using LBC samples from the cervix and endometrium, uterine lavage, urine, and blood for the early diagnosis of ovarian cancer have been conducted. Recently, NGS has advanced remarkably, making the comprehensive detection of genetic mutations in the DNA of tumor tissues possible. FFPE tissues are generally used for NGS analysis; however, the quality of these specimens deteriorates during long-term storage. Therefore, it is preferable to use alcohol-based cytology specimens, which have excellent gene-preservation properties, for NGS analysis. NGS analysis of LBC specimens from patients with endometrial cancer has been reported to be useful in several studies, including the present study (23–25). As a preliminary experiment, ovarian cancer cell lines (SKOV, OVTOKO) were cultured in CelVerse TM as the cell preservation solution and stored refrigerated at 4°C, and the long assay/short assay ratio was calculated using the TaqMan^®^ Assay. Even after approximately 2 years of storage, the L/S ratio was above 0.2, indicating that the samples could be analyzed by the Ion AmpliSeq Cancer Hotspot Panel v2.

In Japan, the proportion of germline variants in ovarian cancer is approximately 18%, and the major genes are *BRCA1, BRCA2*, mismatch repair genes [MutL protein homolog (*MLH*) 1, *MLH2, MLH6*, and postmeiotic segregation increased 2], RAD51 Paralog D, and ataxia telangiectasia mutated (*ATM*) (26). In contrast, *TP53, PIC3CA, KRAS, ARID1A, CTNNB1, SMARCA4, BRCA1, BRCA2*, and *ATR* have been reported as driver genes in ovarian cancer (22).

Of the 19 patients in the present study, one was diagnosed with hereditary breast and ovarian cancer (HBOC). NGS analysis of genomic DNAs in the present study did not include *BRCA*, which is the most common germline variant associated with ovarian cancer. Therefore, we detected only somatic genetic variations and not germline variations. Regarding somatic genetic variation, only *TP53, PIC3CA, KRAS*, and *CTNNB1* were extracted, and no other genetic changes were detected. Among the somatic genetic variants, *TP53* was detected in all HGSC cases. Because HGSC originates from the fallopian tubes, it is assumed that ovarian cancer cells will be detected in the endometrium owing to serous carcinoma lesions in the fallopian tubes. Uterine lavage catheters have been used to detect tumor-specific *TP53* mutations in cells presumably shed from HGSCs (27). In the present study, the detection rate of HGSC was 54.5%, higher than that of other histological types. Because univariate analysis of patients with HGSC showed that the presence of STIC was associated with concordance between cancer tissue sections and LBC specimens, *TP53* is also expected to be detected in early cases, such as STIC cases. Genetic mutations were detected in the LBC specimens from two patients diagnosed with low-chemosensitivity CCC. Identifying the histological type of ovarian cancer before surgery can help determine treatment strategies.

Furthermore, patients with early-stage ovarian cancer were included in the present study. The 5-year survival rate of patients with ovarian cancer at the localized stage is 93.2% (28), which is a good prognosis, and developing a method for early diagnosis of ovarian cancer will contribute to improving the prognosis. Of the six patients at an early stage in the present study, five had 7–35 cm ovarian tumors, whereas one was diagnosed with a normal ovarian size during follow-up at the clinic. Ultrasonography and MRI can be employed to diagnose ovarian cancer. However, two patients with asymptomatic advanced cancer were diagnosed by other departments. Therefore, surveillance methods for the early detection of ovarian cancer, especially in asymptomatic cases, such as HGSC, need to be established. In the present study, early-stage cancer was detected in three cases despite negative cervical and endometrial cytology. Serum microRNA analysis was recently reported as an early diagnostic method for ovarian cancer (29). This method is sufficiently accurate for secondary screening but is costly. Therefore, our proposed method may be useful for efficient primary screening.

This study has some limitations. First, it was a retrospective study with a small sample size. Second, only patients diagnosed with ovarian cancer were included. Therefore, we assessed two patients with benign LBC and did not detect any genetic mutations in their cervical and endometrial LBC specimens. Therefore, asymptomatic patients with ovarian cancer should be evaluated in future studies. We believe that this study will be useful for cases of HBOC, which is considered to have a high incidence, and we are currently investigating the use of our proposed method in patients with HBOC who have not undergone risk-reducing salpingo-oophorectomy. Third, the sensitivity of cervical and endometrial LBC analyses for detecting ovarian cancer was 42%, which was low. Because several somatic genetic variants in ovarian cancer were not included, we intend to create our gene panel test to increase the detection rate. Furthermore, in sampling from the cervix, only one case (5%) was detected, i.e., case 2, and in this case, the mutation was also detected in the endometrium. Although endometrial cytology alone may be sufficient for detection, only cervical cytology is usually performed for cancer screening. Currently, our results indicate that cervical cytology is inadequate as a screening technique for ovarian cancer, but the integration of bioinformatics approaches such as DNA methylation and circulating tumor DNA could increase detection rates. This study may be important as basic data for this analysis.

In conclusion, the detection rate of ovarian cancer using NGS analysis of cervical and endometrial LBC specimens was 42%. It has also been suggested that ovarian cancer has the possibility to be detected at an early stage. The findings of this study can be useful as a noninvasive screening method for detecting somatic mutations associated with ovarian cancer and classifying ovarian cancer.

## Figures and Tables

**Table I.** Clinical data.

Case	Age, years	BMI, kg/m^2^	FIGO stage	Histology	*BRCA* status	HRD status	CA125, U/ml
1	70	23.4	IIIC	Pseudo myxoma	Unknown	Unknown	373.2
2	53	20.8	IC1	CCC	Unknown	Unknown	26.2
3	42	22.6	IC1	CCC	Unknown	Unknown	30.1
4	60	17.7	IC3	CCC	Unknown	Unknown	27.3
5	34	21.1	IC2	MC	Unknown	Unknown	149.1
6	56	17.4	IIIC	MC	Unknown	Unknown	179.4
7	54	29.1	IVB	MC	Unknown	Unknown	139.5
8	80	23.2	IC	EC	Unknown	Unknown	91.9
9	51	30.4	IIA	HGSC	Unknown	Unknown	32.3
10	39	20.5	IIB	HGSC	Negative	Unknown	258.1
11	71	23.7	IIB	HGSC	Unknown	Positive	908.7
12	56	27.0	IIIB	HGSC	Negative	Positive	3,720.8
13	67	21.3	IIIB	HGSC	Negative	Positive	2,455.5
14	70	18.0	IIIC	HGSC	Unknown	Unknown	112.8
15	78	19.2	IIIC	HGSC	Unknown	Positive	5,158.3
16	74	19.0	IIIC	HGSC	Unknown	Negative	2,970.6
17	57	24.1	IVA	HGSC	Unknown	Negative	3,694.4
18	71	26.0	IVB	HGSC	Unknown	Unknown	14.8
19	73	17.6	IVB	HGSC	Positive	Positive	5,632.5

FIGO, International Federation of Gynecology and Obstetrics; HRD, homologous recombination deficiency; CA125, cancer antigen 125; CCC, clear-cell carcinoma; MC, mucinous carcinoma; EC, endometrioid carcinoma; HGSC, high-grade serous carcinoma.

**Table II.** Pathological data.

Case	Ovarian tumor size, cm	Cervical cytology	Endometrial cytology	Ascites cytology	Lymphovascular invasion	STIC	Peritoneal dissemination
1	10	NILM	Negative	Negative	Negative	Presence	Presence
2	7	NILM	Negative	Negative	Negative	Absence	Absence
3	12	NILM	Negative	Negative	Negative	Absence	Absence
4	13	NILM	Negative	Positive	Negative	Absence	Absence
5	35	NILM	Negative	Negative	Negative	Absence	Absence
6	3	NILM	Negative	Positive	Negative	Presence	Presence
7	3	NILM	Negative	Negative	Negative	Absence	Presence
8	16	NILM	Negative	Negative	Negative	Absence	Absence
9	4	NILM	Negative	Negative	Negative	Presence	Absence
10	8	NILM	Negative	Negative	Positive	Absence	Absence
11	3	NILM	AC	Positive	Negative	Absence	Presence
12	6	NILM	Negative	Positive	Negative	Absence	Presence
13	5	NILM	AC	Positive	Negative	Presence	Presence
14	1	AC	AC	Positive	Negative	Presence	Presence
15	12	NILM	Negative	Positive	Negative	Absence	Presence
16	3	NILM	Negative	Positive	Positive	Presence	Presence
17	3	NILM	Negative	Positive	Negative	Absence	Presence
18	4	NILM	Negative	Positive	Negative	Presence	Presence
19	12	NILM	Negative	Negative	Positive	Presence	Presence

AC, adenocarcinoma; NILM, negative for intraepithelial lesion or malignancy; STIC, serous tubal intraepithelial carcinoma.

**Table III.** List of variants of genes in cancer tissue sections and cervical LBC specimens or endometrial LBC specimens.

Gene	Variant	Significance	Case
*TP53*	c.524G>A (p.Arg175His)	Pathogenic	9
	c.814G>A (p.Val272Met)	Pathogenic	11
	c.560-1G>A	Pathogenic	13
	c.818G>A (p.Arg273His)	Pathogenic	14
	c.742C>T (p.Arg248Trp)	Pathogenic	18
	c.659A>G (p.Tyr220Cys)	Pathogenic	19
*PIK3CA*	c.3140A>G (p.His1047Arg)	Pathogenic	2
*RB*	c.2039T>C (p.Ile680Thr)	Pathogenic	4

LBC, liquid-based cytology.

**Table IV.** Univariate analysis of the independent predictors of genetic mutation concordance between cancer tissue sections and liquid-based cytology specimens using a stratified Cox proportional hazards model of all patients.

Variable	HR	95% CI	P-value
Endometrium cytology (negative, n=16; positive, n=3)	3554044654.272	0.640–335.670	0.999
Ascites cytology (negative, n=9; positive, n=10)	2.000	0.312–12.840	0.465
Lymphovascular invasion (negative, n=15; positive, n=4)	1.500	0.164–13.749	0.720
STIC (absence, n=11; presence, n=8)	4.444	0.631–31.294	0.134
Peritoneal dissemination (absence, n=7; presence, n=12)	0.952	0.144–6.281	0.960

STIC, serous tubal intraepithelial carcinoma; HR, hazard ratio.

**Table V.** Univariate analysis of the independent predictors of genetic mutation concordance between cancer tissue sections and liquid-based cytology specimens using a stratified Cox proportional hazards model of patients with high-grade serous carcinoma.

Variable	HR	95% CI	P-value
Endometrium cytology (negative, n=8; positive, n=3)	2692458071.419	0.430–284.320	0.999
Ascites cytology (negative, n=3; positive, n=8)	0.500	0.031–7.994	0.624
Lymphovascular invasion (negative, n=7; positive, n=4)	0.750	0.064–8.834	0.819
STIC (absence, n=5; presence, n=6)	20.000	0.930–429.904	0.056
Peritoneal dissemination (absence, n=2; presence, n=9)	1.250	0.058–26.869	0.887

STIC, serous tubal intraepithelial carcinoma; HR, hazard ratio.

## Data Availability

The NGS data generated in the present study may be found in the DDBJ database under accession number PRJDB19212 or at the following URL: https://ddbj.nig.ac.jp/search/entry/bioproject/PRJDB19212. The other data generated in the present study are included in the figures and/or tables of this article.
